# Domestic Birds as Source of *Cryptococcus deuterogattii* (AFLP6/VGII): Potential Risk for Cryptococcosis

**DOI:** 10.1007/s11046-021-00601-w

**Published:** 2021-11-11

**Authors:** Nathan P. Siqueira, Olívia C. Favalessa, Fernanda H. Maruyama, Valéria Dutra, Luciano Nakazato, Ferry Hagen, Rosane C. Hahn

**Affiliations:** 1 Medical Mycology/Research Laboratory, Medicine School, Federal University of Mato Grosso (UFMT), Fernando Corrêa Avenue, 2387, Boa Esperança, Cuiabá, 78060-900 Brazil; 2 Veterinary Microbiology and Molecular Biology Laboratory, Federal University of Mato Grosso (UFMT), Cuiabá, Brazil; 3 Department of Medical Mycology, Westerdijk Fungal Biodiversity Institute (WI-KNAW), Uppsalalaan 8, 3584CT Utrecht, The Netherlands; 4 Department of Medical Microbiology, University Medical Center Utrecht, Heidelberglaan 100, 3584CX Utrecht, The Netherlands; 5 Mycology Sector, Universitary Hospital Júlio Muller – EBSERH, Cuiabá, Brazil

**Keywords:** *Cryptococcus neoformans*, *Cryptococcus deuterogattii*, *Cryptococcus gattii*, Domestic birds, Excreta, Brazil

## Abstract

Cryptococcosis is an infection caused by encapsulated basidiomycetous yeasts belonging to the *Cryptococcus neoformans/Cryptococcus gattii* species complexes. It is acquired through inhalation of infectious propagules, often resulting in meningitis and meningoencephalitis. The ecological niche of these agents is a wide variety of trees species, as well as pigeon, parrot and passerine excreta. The objective of this study was to isolate *Cryptococcus* yeasts from excreta of commercially traded parrots and passerines. The 237 samples were collected between October 2018 and April 2019 and processed using conventional methodologies. Nineteen colonies with a dark brown phenotype, caused by phenol oxidase activity, were isolated, suggesting the presence of pathogenic *Cryptococcus* yeasts. All isolates tested positive for urease activity. *URA5*-RFLP fingerprinting identified 14 isolates (68.4%) as *C. neoformans* (genotype AFLP1/VNI) and 5 (26.3%) as *C. deuterogattii* (genotype AFLP6/VGII). Multi-locus sequence typing was applied to investigate the relatedness of the *C. deuterogattii* isolates with those collected globally, showing that those originating from bird-excreta were genetically indistinguishable from some clinical isolates collected during the past two decades.

## Introduction

Cryptococcosis is a fungal infection in animals and humans caused by encapsulated basidiomycetous yeasts belonging to the *Cryptococcus neoformans* and *Cryptococcus gattii* species complexes [1, 2]. By inhalation of infectious propagules, the spores, or desiccated yeast cells, reach the pulmonary alveoli and evolve into the pulmonary form of the disease and ultimately spread to the central nervous system causing meningitis or meningoencephalitis [2, 3].

The *C. neoformans* species complex comprises the two species *C. neoformans* (serotype A; genotypes AFLP1/VNI, AFLP1A/VNB/VNII, and AFLP1B/VNII), *C. deneoformans* (serotype D, genotype AFLP2/VNIV) and their hybrids (serotype AD, genotype AFLP3/VNIII) [1]. The *C. gattii* species complex includes five pathogenic species: *C. gattii *sensu stricto (serotype B, genotype AFLP4/VGI), *C. bacillisporus* (serotype B and C, genotype AFLP5/VGIII), *C. deuterogattii* (serotype B, genotype AFLP6/VGII), *C. tetragattii* (serotype C, genotype AFLP7/VGIV) and *C. decagattii* (serotype B, genotype AFLP10/VGVI) [1, 2].

*Cryptococcus* species have been isolated from several ecological niches, such as soil, pigeon droppings and debris in tree holes, and new reservoirs are reported. Environmental sampling attributes to a better understanding of the epidemiology of the disease. The *C. neoformans* species complex has a cosmopolitan distribution and is primarily causing disease in immunocompromised individuals, such as HIV-infected subjects [1, 2]. The *C. neoformans* species complex is associated with organic components in the excreta of pigeons, captive birds, dust and decaying trees of various species [4]. The members of the *C. gattii* species complex are emerging pathogens and were initially considered as ‘tropical and subtropical pathogens’ [5]. However, studies from the past two decades showed that infections and environmental occurrence has expanded to temperate regions, including North America and the north-western part of Europe [6, 7]. In addition, many ecological niches have been investigated worldwide in an attempt to elucidate the environmental reservoirs [7, 8]. In Brazil *C. gattii *sensu lato has been identified from hollow trees [9, 10] and even dust in houses and libraries [11, 12].

Epidemics caused by members of *C. gattii* species complex have been described and ranging from local small outbreaks affecting goats (Spain) [13], sheep (Australia) [14], and parrots in an aviary in São Paulo, Brazil [15]. However, the largest *C. deuterogattii* outbreak so far was first reported early 2000’s from Vancouver Island (B.C. Canada) and has expanded since then to the Pacific Northwest of the U.S.A. [16].

Environmental studies conducted in the state of Mato Grosso, Brazil, have identified *C. neoformans* and *C. gattii* species complexes from different ecological niches [9, 12, 17] as well as clinical isolates from humans and animals [18, 19] contributing to elucidate the epidemiology of cryptococcosis. The aim of this study was to investigate the presence of *Cryptococcus* yeasts in the excreta of captive birds such as parrots and passerines.

## Materials and Methods

### Study Site

Brazil’s third largest state of Mato Grosso (latitude -13° 0′0 S and longitude -56° 0'O) covers an area of 903,206,997 km^2^ in the midwestern region with Cuiabá being its capital city. Mato Grosso, with approximately 3.5 million inhabitants, has three of the Brazilian main ecological systems: The Amazon Forest, Cerrado and Pantanal. The climate is tropical, with rainfall during summer, low humidity during winter, and temperatures ranging from 24 °C to 40 °C [20].

### Sample Collection and Isolation

Two-hundred thirty seven excreta samples were taken from freestanding cages containing a single species of parrots or passerines, collection was performed between October 2018 and April 2019 in Campo Verde, Várzea Grande and Cuiabá. Samples were sent to the Laboratory of Medical Mycology/Research at the Federal University of Mato Grosso (UFMT), where processing was performed according to the protocols described previously by Filiú and co-workers [21] and Lazéra and colleagues [22] with few modifications. Briefly, two grams of the sample was suspended in 8 mL of distilled water and 0.4 g of chloramphenicol was added. The samples were thereafter homogenized and allowed to settle for 1 h. From each sample, 100 µL of the supernatant was seeded onto ten Niger Seed Agar (NSA) plates that were supplemented with chloramphenicol (0.4 g/L) and amikacin (120 µl/L). Media were incubated at 35 °C in a biological oxygen demand incubator for 72 h. Thereafter, media were examined for brown colonies suggestive of being members of the *C. neoformans/C. gattii* species complexes*.* All isolate with characteristics suggestive for being *Cryptococcus* were inoculated onto urea medium and incubated at 35 °C in a biological oxygen demand incubator for 72 h and examined [23].

### Molecular Characterization

DNA extraction was performed according to the protocol described by Del Poeta and colleagues [24].

DNA was stored at –20 °C until further use. Cryptococcal isolates were genotyped using *URA5*-RFLP according to the methodology described by Meyer and co-workers [25]. The *URA5* amplicons were overnight digested at 37 °C with Sau96I (10 µl) and Hhal (20 µl) endonucleases (New England Biolabs), fingerprints were visualized onto a 3% agarose gel that included the reference strains for each of the molecular types [25]. Isolates that were identified as *C. deuterogattii* were further molecularly investigated by multi-locus sequence typing (MLST) as previously described [26]. Obtained sequence-data was added to a reference set of *C. deuterogattii* MLST data representing all known sequence types for this species, subsequently phylogenetic analysis was performed in MEGA v7 using the maximum likelihood method as previously described [26–28].

## Results

Among the 237 collected excreta samples, 142 (59.9%) originated from the order of parrots and 95 (40.1%) from passerines, comprising 16 genera and 17 species. The majority of samples came from cockatiels (*Nymphicus hollandicus*) (n = 71/237), followed by the Atlantic canaries (*Serinus canarius*) (n = 50/237) and budgerigars (*Melopsittacus undulatus*) (n = 33/237). Samples were collected in the capital Cuiabá (175/237; 73.8%), 39/237 (16.5%) in Campo Verde and 23/237 (9.7%) in Várzea Grande (Table 1).

**Table 1:** Distribution of excreta samples according to parrot and passerine species

Bird	Order	Species name	Total	Percentage
Cockatiel	Psittaciformes	*Nymphicus hollandicus*	71	30
Atlantic canary	Passeriformes	*Serinus canarius*	50	21
Budgerigar	Psittaciformes	*Melopsittacus undulatus*	33	11
Lovebird	Psittaciformes	*Agapornis* species	23	10
Ring-necked parakeet	Psittaciformes	*Psittacula krameri*	13	6
Eastern rosella	Psittaciformes	*Platycercus eximius*	7	4
Grey parrot	Psittaciformes	*Psittacus erithacus*	6	2.6
Gouldian finch	Passeriformes	*Erythrura gouldiae*	6	2.6
Zebra finch	Passeriformes	*Taeniopygia guttata*	6	2.6
Java sparrow	Passeriformes	*Padda oryzivora*	4	1.8
Society finch	Passeriformes	*Lonchura striata domestica*	4	1.7
Bourke’s parrot	Psittaciformes	*Neopsephotus bourkii*	4	1.7
Red-rumped parrot	Psittaciformes	*Psephotus haematonotus*	3	1.5
American kestrel	Falconiformes	*Falco sparverius*	2	1
Alexandrine parakeet	Psittaciformes	*Psittacula eupatria*	2	1
Rock dove	Columbiformes	*Columba livia*	1	0.5
Plum-headed parakeet	Psitaciformes	*Psittacula cyanocephala*	1	0.5
Barred parakeet	Psittaciformes	*Bolborhynchus lineola*	1	0.5
Total	–	–	237	100

**Fig. 1:**
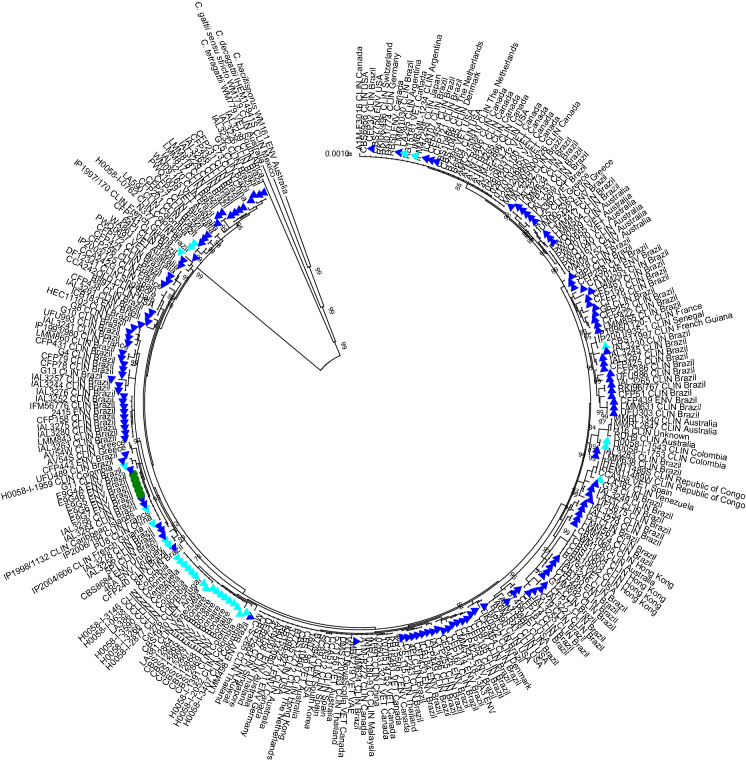
Multi-locus sequence typing-based phylogenetic analysis of *Cryptococcus deuterogattii* isolates. Maximum likelihood phylogenetic analysis was performed in MEGA v7 using settings as previously described [26–28]. The tree with the highest log likelihood (− 12,291.85) is shown. Initial tree(s) for the heuristic search were obtained automatically by applying Neighbor-Join and BioNJ algorithms to a matrix of pairwise distances estimated using the Maximum Composite Likelihood (MCL) approach, and then selecting the topology with superior log likelihood value. A discrete Gamma distribution was used to model evolutionary rate differences among sites (5 categories (+ G, parameter = 0.1000)). The rate variation model allowed for some sites to be evolutionarily invariable ([+ I], 43.83% sites). The tree is drawn to scale, with branch lengths measured in the number of substitutions per site. The analysis involved 270 nucleotide sequences. All positions containing gaps and missing data were eliminated. There were a total of 3997 positions in the final dataset. The five *C. deuterogattii* isolates from the current study are indicated with a green circle, other Brazilian *C. deuterogattii* isolates reported previously are demarcated with a dark blue triangle, while bright blue triangles indicate isolates that originated from other Latin American countries. Isolates WM161, IHEM14941S, WM179 and WM779 served as outgroup, representing *C. bacillisporus*, *C. decagattii*, *C. gattii *sensu stricto and *C. tetragattii* reference strains, respectively

Out of the 237 samples collected and plated onto NSA, 19 (8.0%) isolates showed morphological characteristics suggestive for members of the *C. neoformans/C. gattii* species complexes, as they had dark brown coloration of the colonies due to phenol oxidase production. These isolates were all urease positive. The molecular types of the isolates were determined by *URA5*-RFLP. Fourteen out of nineteen isolates (73.2%) were *C. neoformans *sensu stricto molecular type VNI (= genotype AFLP1/VNI) and five (26.3%) were *C. deuterogattii* molecular type VGII (= genotype AFLP6/VGII). For *C. neoformans *sensu stricto, cockatiels (*N. hollandicus*) showed a greater number of positive results (n = 12, 85.6%), followed by Red rumped parrots (*P. haematonotus*) and the Atlantic canary (*S. canarius*), both represented by one isolate (each 7.2%). On the other hand, *C. deuterogattii* was obtained from cockatiels (*N. hollandicus*) excreta (n = 4; 80%) and Bourke’s parrot (*N. bourkii*) excreta (n = 1; 20%). The maximum likelihood phylogenetic analysis of the MLST data showed that all five *C. deuterogattii* isolates were genetically indistinguishable from each other and from clinical isolates collected up to 2 decades ago from Brazil, Caribbean Islands, France, French Guiana and China (Fig. 1). Sequences were deposited in NCBI GenBank under accession numbers MZ393809-MZ393843 (Table 2).

**Table 2:** Distribution of *Cryptococcus neoformans*/*Cryptococcus gattii* species complexes isolated from bird excreta in commercial establishments

Sample ID* (CFP accession nr.)^#^	Popular name	Scientific name	*Cryptococcus* species (molecular type)
E5G2 (CFP00952)	Atlantic canary	*S. canarius*	*C. neoformans* (VNI)
E9G4A (CFP00984)	Cockatiel	*N. hollandicus*	*C. deuterogattii* (VGII)
E9G4B (CFP00985)	Cockatiel	*N. hollandicus*	*C. deuterogattii* (VGII)
E9G5 (CFP00986)	Bourke’s parrot	*N. bourkii*	*C. deuterogattii* (VGII)
E9G6 (CFP00953)	Cockatiel	*N. hollandicus*	*C. deuterogattii* (VGII)
E9G7 (CFP00954)	Cockatiel	*N. hollandicus*	*C. deuterogattii* (VGII)
E9G8 (CFP00955)	Cockatiel	*N. hollandicus*	*C. neoformans* (VNI)
E9G9 (CFP00956)	Cockatiel	*N. hollandicus*	*C. neoformans* (VNI)
E9G11 (CFP00957)	Cockatiel	*N. hollandicus*	*C. neoformans* (VNI)
E9G14 (CFP00958)	Cockatiel	*N. hollandicus*	*C. neoformans* (VNI)
E9G15 (CFP00959)	Cockatiel	*N. hollandicus*	*C. neoformans* (VNI)
E9G16 (CFP00960)	Red rumped parrot	*P. haematonotus*	*C. neoformans* (VNI)
E9G17 (CFP00961)	Cockatiel	*N. hollandicus*	*C. neoformans* (VNI)
E9G19 (CFP00962)	Cockatiel	*N. hollandicus*	*C. neoformans* (VNI)
E9G20 (CFP00963)	Cockatiel	*N. hollandicus*	*C. neoformans* (VNI)
E9G21 (CFP00987)	Cockatiel	*N. hollandicus*	*C. neoformans* (VNI)
E9G24 (CFP00988)	Cockatiel	*N. hollandicus*	*C. neoformans* (VNI)
E9G34 (CFP00989)	Cockatiel	*N. hollandicus*	*C. neoformans* (VNI)
E9G35 (CFP00990)	Cockatiel	*N. hollandicus*	*C. neoformans* (VNI)

*E = Establishment/store; G = collection cage

^#^ = FIOCRUZ culture collection accession number

## Discussion

The natural habitat of members of the *C. neoformans and C. gattii* species complexes has been extensively studied, especially in areas where the incidence of cryptococcosis is relatively high [5, 28]. The primary ecological niche of *C. neoformans *sensu stricto was repetitively found to be bird excreta, especially pigeon excreta. Three decades ago, *C. gattii *sensu lato was isolated from plant debris under a *Eucalyptus camaldulensis* tree in Australia [29]. The distribution pattern of *E. camaldulensis* was associated with the relatively high proportion of cryptococcal infections among rural aboriginals, compared to other areas [30].

Since then, the investigation of *C. neoformans/C. gattii* species complex members has been carried out in a large variety of niches, which repetitively showed that *C. neoformans *sensu stricto was mostly isolated from bird excreta while *C. gattii *sensu lato has been associated with tree/plant debris [9–13].

In Brazil, isolation of *Cryptococcus* yeast species from captive birds’ excreta has been reported from the states of Paraná, Rio de Janeiro, Pará and Mato Grosso do Sul [17, 21, 31–35]. While analyzing the number of isolates obtained in the current study, a greater number of positive samples were observed from cockatiel excreta (*N. hollandicus*). This species is popular, and more expensive, among the commercialized birds. In contrast, Pereira and colleagues [35] reported a low isolation rate for this species while more isolates were obtained from the excreta of the budgerigar (*Melopsittacus undulatus*). On the other hand, Lugarini and co-workers [32] obtained a higher number of isolates from Saffron finches (*Sicalis flaveola*) and it was observed that the yeasts may be distributed via excreta of Psittaciformess and Passeriformess regardless of the bird species.

In the current study, 13 out of 19 (72.2%) isolates were *C. neoformans *sensu stricto and were isolated from Psittaciformes and Passeriformes excreta. This corroborates results from the studies by Lugarini and colleagues [32] performed in the state of Paraná where 25.5% (n = 36/141) were *C. neoformans *sensu stricto, and Passoni and co-workers [33] performed in the city of Rio de Janeiro where 4.3% (n = 54/1,268) of the samples were *C. neoformans *sensu lato positive. Other studies have shown higher numbers of *C. neoformans *sensu stricto isolates from pigeon excreta in the city of Belém [31], and Campo Grande [21]. In Cuiabá, *C. neoformans *sensu stricto was also identified, mostly from niches such as pigeon excreta at various locations in the city as reported by Takahara and co-workers [17].

Cryptococcosis, an opportunistic fungal infection, is often diagnosed among HIV/AIDS patients, and is also a major cause of morbidity and mortality, with *C. neoformans *sensu stricto molecular type VNI being the most prevalent worldwide among clinical and environmental strains [1, 35]. This holds true for Brazil, where *C. neoformans *sensu stricto VNI predominates in clinical isolates among HIV-infected patients, mainly in the Southern, Southeastern and Midwestern regions [4, 36, 37].

It was believed that the River red gum tree (*Eucalyptus camaldulensis*) was the niche of *C. gattii *sensu lato, consequently others investigated these trees in other countries such as India, United States, Mexico [30, 38, 39]. That only *E. camaldulensis* was the single niche for *C. gattii *sensu lato has been disputed successfully by others. For example, Lazéra and colleagues reported the isolation of two cryptococcal species, *C. deneoformans* (cited as *C. neoformans* var. *neoformans*) and *C. gattii *sensu lato (cited as *C. neoformans* var. *gattii*) from a single *Cassia javanica* tree in the city of Teresina (Brazil). And large-scale environmental screening for *C. deuterogattii* in British Columbia (Canada) showed that (decaying) trees in general are the primary niche [8, 40].

*Cryptococcus gattii *sensu lato is rarely isolated from excreta of captive birds, however, the present study observed 26.3% (n = 5/19) positive samples harbouring *C. deuterogattii*. Which differs from the study by Abegg and colleagues who isolated *C. gattii *sensu stricto (genotype AFLP4/VGI) from bird excreta [41]. *Cryptococcus deuterogattii* in this study came from samples collected from cockatiel excreta at two locations, namely a veterinary clinic and from a small farm. From the latter location samples were collected from six tree holes near the bird cages, but these samples yielded no cryptococcal growth. A case cluster caused by *C. gattii *sensu lato yeasts has been reported to have affected Psittaciformes species in an aviary zoo in the state of São Paulo [15]. Yeasts from the nasal region, excreta and liver were isolated from one of the birds and identified as serotype B [15] and subsequently confirmed by molecular tools as being *C. deuterogattii* [42].

Studies conducted in the city of Cuiabá showed the presence of *C. deuterogattii*, the agent causing the ongoing and expanding outbreak on Vancouver Island [41], in library dust [12], in tree holes (*Plathymenia reticulata*) located in the central urban area of the city [9], in clinical isolates mostly from immunocompetent patients and also from HIV/AIDS patients [4]. The current study records the first isolation of *C. deuterogattii* from Psittaciformes and Passeriformes excreta.

Other environmental studies that reported isolation of bird-associated *C. gattii *sensu lato isolates [15, 21, 31, 32, 41], obtained cryptococcal isolates from excreta but it could not be excluded that the excreta was from other bird species. The isolation of members of the *C. neoformans/C. gattii* species complexes from bird excreta does not mean that a particular bird species has a specific role as a reservoir. Nevertheless, it indicates that bird excreta contributes to the aerial dispersion of infectious *Cryptococcus* propagules, allowing its transmission to humans and other mammals [43]. Captive bird breeders' staff, who frequently perform daily cage cleaning and bird grooming, may be exposed to high concentrations of infectious propagules that enables the acquisition of a cryptococcal infection.
